# Supplementary material for: Mitochondrial Oxidative Stress Induces Cardiac Fibrosis in Obese Rats through Modulation of Transthyretin
Source: Int J Mol Sci. 2022 Jul 22;23(15):8080. doi: 10.3390/ijms23158080 (PMC9330867; doi:10.3390/ijms23158080)

Figure S1: Original images obtained from western blot presented in Figure 4:

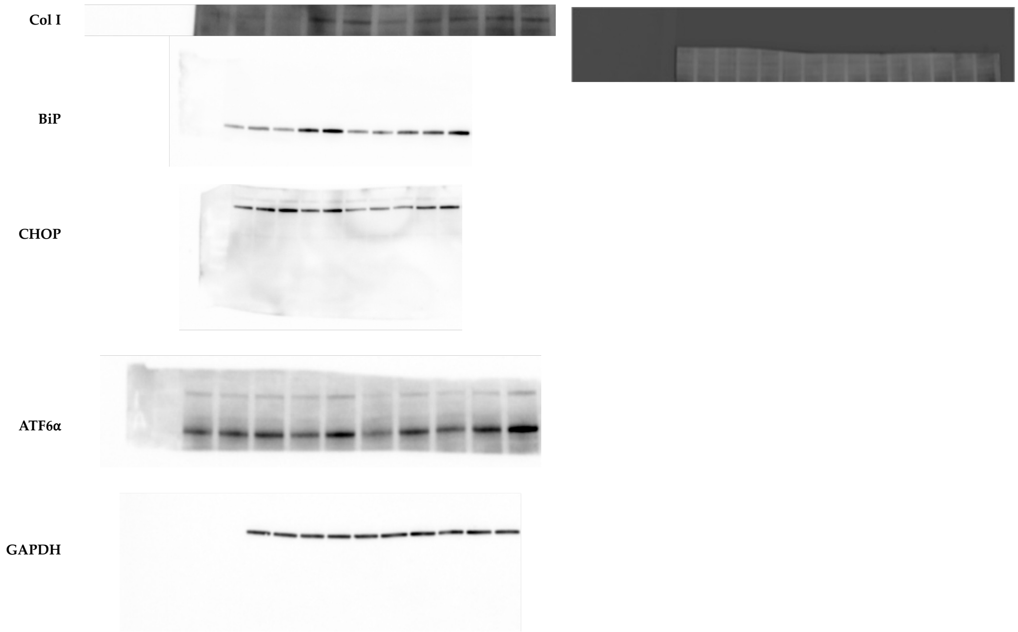

Figure S2: Original images obtained from western blot presented in Figure 5:

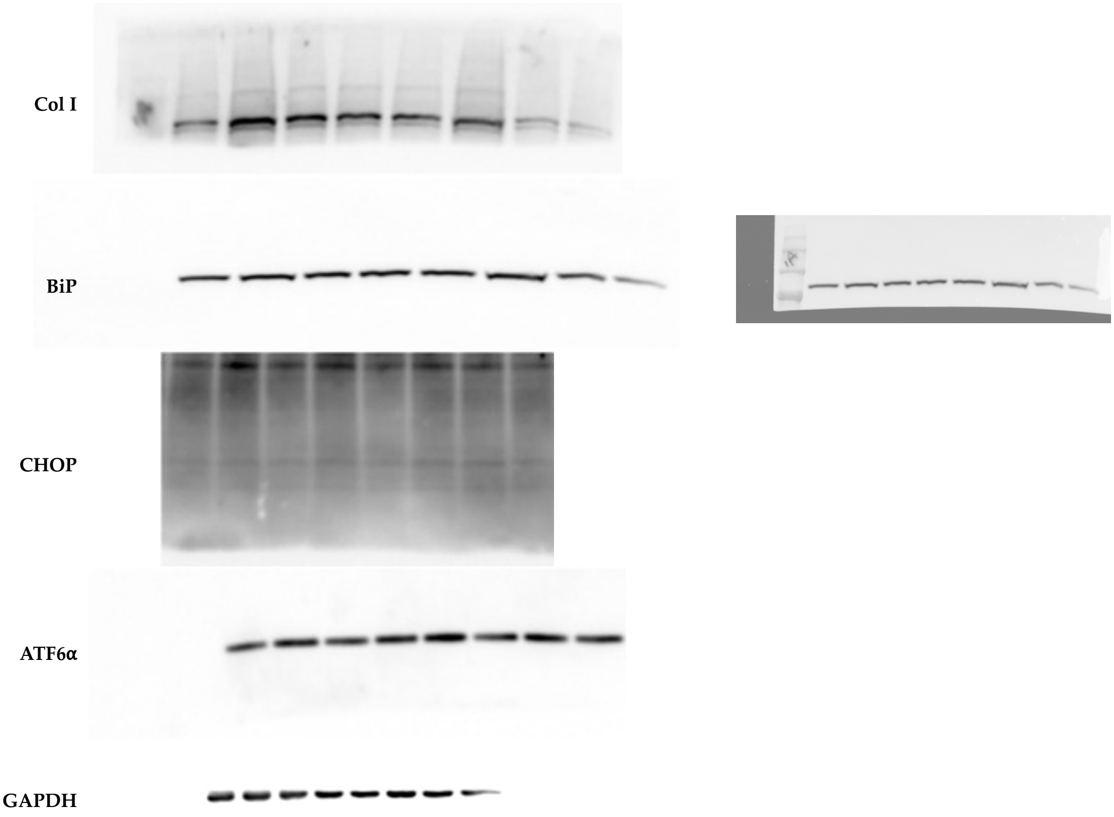

Supplement: Supplementary file 1 [file ijms-23-08080-s001.zip › Supplemental Figures S1 and S2.pdf]
