# Supplementary material for: Mitochondrial Oxidative Stress Induces Cardiac Fibrosis in Obese Rats through Modulation of Transthyretin
Source: Int J Mol Sci. 2022 Jul 22;23(15):8080. doi: 10.3390/ijms23158080 (PMC9330867; doi:10.3390/ijms23158080)
Supplement: Supplementary file 1 [file ijms-23-08080-s001.zip › Supplemental Table S1, Table S2 and Table S4.pdf]

Table S1: Differential cardiac expressed proteins modulated by obesity.

Protein names	Gene names	Uniprot	Peptide count	p-Value
Protein disulfide-isomerase A6	PDIA6	A0A0G2JSZ5	6	0.000
Sorbin and SH3 domain-containing protein 1	SORBS1	F1M8Z8	19	0.002
Nucleolin	NCL	Q5U328	3	0.005
Cytochrome b-c1 complex subunit 8	UQCRQ	Q7TQ16	2	0.003
Peroxisomal multifunctional enzyme type 2	HSD17B4	P97852	9	0.003
Histone H2A	H2AFX	D3ZXP3	3	0.004
Proteasome subunit alpha type	PSMA7	A0A0G2K0W9	2	0.008
Isocitrate dehydrogenase [NAD] subunit, mitochondrial	IDH3G	Q5XIJ3	8	0.019
Phosphatidate cytidylyltransferase	CDS2	G3V8W2	2	0.014
14-3-3 protein eta	YWHAH	P68511	5	0.013
Apolipoprotein A-IV	APOA4	P02651	8	0.035
Protein Scrn3	SCRN3	A0A0G2K189	3	0.042
Integrin-linked protein kinase	ILK	Q99J82	5	0.033
Protein Hccs	HCCS	D3ZL85	2	0.001
Transthyretin	TTR	P02767	2	0.023
Vesicle-associated membrane protein-associated protein B	VAPB	Q9Z269	4	0.007
Four and a half LIM domains protein 2	FHL2	O35115	15	0.037
Protein Vtn	VTN	Q3KR94	2	0.003
Myosin-11	MYH11	E9PTU4	11	0.047
Protein Hist1h2bf	HIST1H2BL	M0R4L7	3	0.010
Cathepsin B	CTSB	Q6IN22	3	0.025
Tryptophan--tRNA ligase, cytoplasmic	WARS	F8WFH8	2	0.007
Chaperonin containing Tcp1, subunit 6A (Zeta 1)	CCT6A	Q3MHS9	9	0.022
Cysteine conjugate-beta lyase 1, isoform CRA_a	CCBL1	G3V827	3	0.044
Purine nucleoside phosphorylase	PNP	D3ZXK9	3	0.020
Keratin, type II cytoskeletal 1	KRT1	A0A0G2JST3	6	0.042
Protein Twf2	TWF2	B0BMY7	2	0.037
Mitochondrial pyruvate carrier 2	MPC2	P38718	4	0.033
Glycogen [starch] synthase, muscle	GYS1	A2RRU1	10	0.048
Serine hydroxymethyltransferase	SHMT2	Q5U3Z7	4	0.041
NADH-ubiquinone oxidoreductase chain 2	MTND2	P11662	3	0.016
Lysine--tRNA ligase	KARS	Q5XIM7	6	0.018
Valine--tRNA ligase	VARS	Q04462	5	0.023

Table S2: Differential cardiac expressed proteins modulated by mitochondrial oxidative stress in obesity.

Protein names	Gene names	Uniprot	Peptide count	p-Value
Cytochrome b-c1 complex subunit 8	UQCRQ	Q7TQ16	2	0.019
Phosphatidate cytidylyltransferase	CDS2	G3V8W2	2	0.028
14-3-3 protein eta	YWHAH	P68511	5	0.035
Protein Scrn3	SCRN3	A0A0G2K189	3	0.045
Transthyretin	TTR	P02767	2	0.042
40S ribosomal protein S4, X isoform	RPS4X	A0A0H2UHX3	2	0.028
Carbonic anhydrase 1	CA1	B0BNN3	11	0.049
Cytosolic non-specific dipeptidase	CNDP2	Q6Q0N1	5	0.012
Hsp90 co-chaperone Cdc37	CDC37	Q63692	5	0.045
5'-nucleotidase	NT5C3A	B2GUX5	2	0.021
Acid ceramidase	ASAH1	A0A0G2K8T0	4	0.023
Protein Sfpq	SFPQ	A0A0G2K8K0	2	0.036
LanC-like protein 1	LANCL1	Q9QX69	4	0.000
C-reactive protein	CRP	P48199	4	0.001
Staphylococcal nuclease domain-containing protein 1	SND1	D4A8Y5	4	0.038
Thioredoxin reductase 2, mitochondrial	TXNRD2	F1M6X5	5	0.024
Heat shock protein 75 kDa, mitochondrial	TRAP1	Q5XHZ0	13	0.039
Protein Synpo2l	SYNPO2L	D3ZZ68	2	0.006
Peroxisomal bifunctional enzyme	EHHADH	P07896	2	0.022
Protein-glutamate O-methyltransferase	ARMT1	Q6AYT5	3	0.004
Basal cell adhesion molecule	BCAM	Q9ESS6	6	0.000
Protein LOC102554591	LOC102554591	A0A0G2JWS2	28	0.011
Protein Acacb	ACACB	A0A0G2K1F2	8	0.009

Table S4: Diet composition used in the study.

	Control diet	High fat diet
**Protein (% by weight)**	14.3	20.4
**Carbohydrate (% by weight)**	48.0	36.1
**Fat (% by weight)**	4.0	35.2
**kcal/g**	2.9	5.4
**% kcal from protein**	20.0	15.0
**% kcal from carbohydrate**	67.0	26.6
**% kcal from fat**	13.0	58.4
